# Weight trajectories throughout adulthood and prostate cancer incidence, aggressiveness, and death in 258 494 men

**DOI:** 10.1093/jnci/djag014

**Published:** 2026-01-23

**Authors:** Marisa da Silva, Josef Fritz, Ahmed Elhakeem, Sylvia H J Jochems, Ming Sun, Innocent B Mboya, Christel Häggström, Jens Wahlström, Karl Michaëlsson, Patrik K E Magnusson, Ylva T Lagerros, Lena Lönnberg, Abbas Chabok, Sölve Elmståhl, Bright I Nwaru, Hannu Kankaanranta, Linnea Hedman, Helena Backman, Sara Hägg, Pär Stattin, Kate Tilling, Tanja Stocks

**Affiliations:** Department of Translational Medicine, Lund University, Malmö, Sweden; School of Information Technology, Halmstad University, Halmstad, Sweden; Department of Translational Medicine, Lund University, Malmö, Sweden; Institute of Clinical Epidemiology, Public Health, Health Economics, Medical Statistics and Informatics, Medical University of Innsbruck, Innsbruck, Austria; Population Health Sciences, Bristol Medical School, University of Bristol, Bristol, United Kingdom; MRC Integrative Epidemiology Unit, University of Bristol, Bristol, United Kingdom; Institute for Risk Assessment Sciences, Utrecht University, Utrecht, The Netherlands; Department of Translational Medicine, Lund University, Malmö, Sweden; Department of Translational Medicine, Lund University, Malmö, Sweden; Africa Academy for Public Health, Dar es Salaam, Tanzania; Department of Epidemiology and Biostatistics, Institute of Public Health, Kilimanjaro Christian Medical University College, Moshi, Tanzania; Northern Registry Centre, Department of Diagnostics and Intervention, Umeå University, Umeå, Sweden; Department of Epidemiology and Global Health, Umeå University, Umeå, Sweden; Medical Epidemiology, Department of Surgical Sciences, Uppsala University, Uppsala, Sweden; Department of Medical Epidemiology and Biostatistics, Karolinska Institutet, Stockholm, Sweden; Department of Medicine, Huddinge, Karolinska Institutet, Stockholm, Sweden; Centre for Clinical Research, Västmanland, Uppsala University, Västerås, Sweden; Department of Clinical Sciences, Division of Surgery, Danderyd Hospital, Karolinska Institutet, Stockholm, Sweden; Department of Clinical Sciences Malmö, Lund University, Malmö, Sweden; Krefting Research Centre, Department of Internal Medicine and Clinical Nutrition, Institute of Medicine, Sahlgrenska Academy, University of Gothenburg, Gothenburg, Sweden; Wallenberg Centre for Molecular and Translational Medicine, University of Gothenburg, Gothenburg, Sweden; Krefting Research Centre, Department of Internal Medicine and Clinical Nutrition, Institute of Medicine, Sahlgrenska Academy, University of Gothenburg, Gothenburg, Sweden; Department of Respiratory Medicine, Seinäjoki Central Hospital, Seinäjoki, Finland; Faculty of Medicine and Health Technology, Tampere University, Tampere, Finland; Department of Public Health and Clinical Medicine, The OLIN and Sunderby Research Unit, Umeå University, Umeå, Sweden; Department of Public Health and Clinical Medicine, The OLIN and Sunderby Research Unit, Umeå University, Umeå, Sweden; Department of Medical Epidemiology and Biostatistics, Karolinska Institutet, Stockholm, Sweden; Department of Surgical Sciences, Uppsala University, Uppsala, Sweden; Population Health Sciences, Bristol Medical School, University of Bristol, Bristol, United Kingdom; MRC Integrative Epidemiology Unit, University of Bristol, Bristol, United Kingdom; Department of Translational Medicine, Lund University, Malmö, Sweden

## Abstract

**Background:**

Obesity assessed at a single time point in adulthood has shown no consistent association with prostate cancer (PCa) incidence but has been positively associated with PCa death. We investigated the association of total and age-specific adult weight trajectories with PCa aggressiveness and death.

**Methods:**

We analyzed data from 258,494 men in Sweden with at least 3 weight observations between ages 17 and 60. Individual weight trajectories were estimated using linear mixed-effects models with natural cubic and linear splines for age, incorporating random intercepts and slopes. These estimates were included in multivariable-adjusted Cox proportional hazards models.

**Results:**

Over a median follow-up of 25 years, 22 055 men were diagnosed with PCa and 4547 died from the disease. Steep weight gain was inversely associated with PCa diagnosed during the PSA testing era (1997 onwards) and via asymptomatic PSA testing, but not with aggressive PCa. Among men with PCa, steep weight gain was associated with increased risk of PCa death (HR quintile 5 vs. 1 = 1.23, 95% CI = 1.08 to 1.40), primarily driven by weight gain between ages 45 and 60 (HR per 1 kg/year = 1.31, 95% CI = 1.10 to 1.57).

**Conclusions:**

The associations observed for incident PCa appear to be influenced by PSA testing uptake; however, the extent to which detection bias contributes remains uncertain. Conversely, late midlife weight gain was associated with an elevated risk of PCa death, underscoring the importance of weight management during this period as a potentially modifiable factor for reducing PCa death.

## Introduction

Obesity is an established risk factor for numerous diseases, including metabolic and cardiovascular disorders, as well as certain cancers.^1^^,^^2^ Most of this evidence is derived from anthropometric data collected once, typically in middle to late adulthood. Prostate cancer (PCa) is the second most diagnosed cancer in men globally and the most diagnosed in Sweden.^3^ Recent systematic evidence on the relationship between obesity and cancer has not identified obesity as a risk factor for PCa incidence; however, some evidence suggests an association between obesity and PCa death.^4^^,^^5^ Subsequent large-scale prospective studies and Mendelian randomization studies^6-9^ have demonstrated an inverse association between obesity and non-aggressive PCa^6^^,^^7^ while showing no evidence of an association with aggressive PCa.^6-9^ In the late 1990s, Sweden adopted widespread prostate-specific antigen (PSA) testing, delineating a pre-PSA and PSA era. The adoption of PSA testing led to a notable increase in low-risk, or non-aggressive, PCa.^10^^,^^11^ Data from the UK Biobank has shown that men with a generally healthy lifestyle, eg, of normal weight and non-smokers, are more likely to undergo asymptomatic PSA testing that primarily detects non-aggressive PCa.^12^ This has posed challenges in identifying risk factors with an unbiased association with PCa.

Changes in body weight throughout the life course may influence disease risk independently of baseline body weight.^13^ Body mass index (BMI) combines fat mass and lean body mass, whereas weight change in adults more accurately reflects body fat.^14^ An increase in weight during adulthood, particularly in late adulthood, typically indicates an increase in fat mass rather than lean mass. Adiposity assessed at various time points and body weight changes over the life course may differentially affect the potential association with PCa.^15^ A limited number of studies have investigated the relationship between weight trajectories and PCa.^16-20^ Among these studies, 2 studies based on the same US cohort included repeated weight assessments,^16^^,^^18^ as opposed to other studies of weight change that relied on self-reported recalled weight and current weight at baseline. These same 2 studies included PCa diagnosed both before and in the PSA era; however, the results were not stratified by these time periods, which has been employed in a few studies examining the association between BMI and PCa.^6^^,^^21^ Evidence suggests that weight gain is inversely associated with non-aggressive PCa,^22^ while it is positively associated with aggressive PCa^17^^,^^19^ and PCa death.^7^^,^^17^ To our knowledge, no study has distinguished weight trajectories in different age periods associated with PCa. This study aimed to elucidate the association between weight trajectories throughout adulthood (ages 17-60) and the incidence and death from PCa and to further examine these associations within specific age intervals, stratified by cancer aggressiveness, PSA eras, and the mode of cancer detection.

## Methods

### Study design and participants

The Obesity and Disease Development Sweden (ODDS) study is a pooled cohort with individual-level data on weight and height from Swedish research cohorts and nationwide registers.^23^ This study included 258 494 men with 1 226 021 cancer-free weight measurements, originating from those with at least 3 weight measurements between the ages of 17 and 60 years, recorded between 1963 and 2019. Details of the applied exclusions are illustrated in Figure S1 (see online supplementary material for a color version of this figure). Most weight observations (69%) were obtained from the Construction Workers Cohort, which collected objectively measured weight and height during routine health examinations conducted between 1971 and 1993. These examinations were offered to nearly all construction workers in Sweden. Other major sources included 2 population-based cohorts, the Cohort of Swedish Men from Västmanland and Örebro counties and the Northern Sweden Health and Disease Study, as well as the Military Conscription Register, which comprised men conscripted primarily before 2010, when conscription was mandatory. An overview of all included cohorts is provided in Table S1 (see online supplementary material). The Swedish Ethical Review Authority approved the study (No: 2020-03846).

### Record and register linkages

Participants were followed using the unique personal identity number assigned to each resident of Sweden.^24^ This enabled longitudinal tracking of repeated measurements of weight across cohorts (including overlapping participation), as well as virtually complete linkage of demographic data from the Total Population Register^25^ and sociodemographic data from the Longitudinal Integrated Database for Health Insurance and Labour Market Studies (LISA).^26^ Cancer diagnoses were obtained from the Cancer Register,^27^ while diagnostic PCa information and reasons for diagnostic work-up were retrieved from the National Prostate Cancer Register.^28^ Date and the primary cause of death were retrieved from the Cause of Death Register.^29^

### Exposure and outcome assessment

Weight data (kg) were collected through 3 modes: objectively measured (83%), self-reported current (12%), and self-reported historical (5%). Only weight observations recorded prior to any cancer diagnosis other than non-melanoma skin cancer were retained, with all post-diagnosis observations excluded from the dataset. Individual trajectories were modelled using linear mixed-effects (LME) models with natural cubic splines (4 knots) and linear splines for age, incorporating measurement mode as a fixed effect.^30^ Random effects included best linear unbiased predictions (BLUPs) of individual intercepts and slopes. Model specification was guided by graphical assessment of linear and polynomial age terms. Natural cubic splines captured overall weight trajectories, while linear splines provided interpretable estimates of weight change (kg/year) across age intervals. No substantial differences were observed between linear and polynomial models when compared with observed and specific age period trajectories. Individual weight slopes were categorized into quintiles and mean predicted weights by age within each quintile were calculated for visualization. For age-specific analysis, trajectories were segmented into 3 periods, 17 to <30, 30 to <45, and 45 to 60 years, to estimate growth rates within each ∼15-year interval. Outcomes included total, non-aggressive, and aggressive incident PCa, and PCa death. Aggressive PCa was defined as T4, N1, M1, Gleason score ≥8, or a PSA level of ≥50 ng/mL at diagnosis.^31^ Non-aggressive PCa encompassed all other cases.

### Statistical analysis

We used a 2-stage approach to estimate hazard ratios (HR) and 95% confidence intervals (CI) for the association between weight trajectories and PCa outcomes.^32^^,^^33^ In the first stage, we fitted LME models to estimate individual weight trajectories (intercept and slope), as previously described. In the second stage, we included the quintiles and individual-specific random weight slopes in Cox proportional hazards models, with age as the underlying timescale. Participants were followed from the date of their last weight observation until a cancer diagnosis, emigration, death, or end of follow-up, December 31, 2019, whichever occurred first. Due to the long follow-up time and changed PCa incidence over time, all Cox models included year of birth as strata (<1930, 1930 to <1945, 1945 to <1960, ≥1960) and were adjusted for age, predicted weight at age 17 (continuous), and height (continuous). Additionally, adjustments were made for highest educational attainment, marital status, and country of birth for individuals and their parents as categorized in Table 1. Models for PCa death were further adjusted for smoking status (never, former, current, missing; 1-3% missingness across weight trajectory quintiles). Smoking was included only in PCa death analyses, reflecting prior evidence of its association with death but not incidence.^34^ Age period models were further adjusted for random weight slopes from preceding age intervals, thereby accounting for longitudinal trends prior to each respective age period.

**Table 1.** Descriptive characteristics of 258 494 men in the Obesity and Disease Development Sweden study (1963-2019) with at least three cancer-free weight observations between ages 17 and 60.

	Adult weight trajectories in quintiles (ages 17 to 60 years)				
	Q1	Q2	Q3	Q4	Q5
Study and population characteristics					
No. of men	51 699	51 699	51 699	51 699	51 698
No. of obs.	257 651	245 954	236 250	238 163	248 003
No. of obs./man, median (IQR)	5 (4-7)	5 (4-7)	5 (3-7)	5 (4-7)	5 (4-7)
Time from first to last weight obs. (years), median (IQR)	17 (12-28)	16 (10-21)	15 (9-20)	16 (9-21)	17 (12-28)
Predicted weight change (kg), mean (SD)	6.5 (5.2)	14.1 (1.5)	18.8 (1.3)	23.7 (1.7)	34.9 (8.2)
Predicted weight change (kg), median (IQR)	7.7 (4.7-9.8)	14.2 (12.9-15.4)	18.8 (17.7-19.9)	23.6 (22.3-25.1)	32.5 (29.3-37.8)
Mode of weight measurement, % (obs.)					
Objectively measured	76.9 (198 178)	81.7 (200 947)	84.7 (200 199)	85.9 (204 624)	84.9 (210 511)
Self-reported current	5.8 (14 830)	4.9 (12 038)	4.3 (10 109)	4.2 (9970)	4.5 (11 281)
Self-reported historic	17.3 (44 643)	13.4 (32 969)	11.0 (25 942)	9.9 (23 569)	10.6 (26 211)
Year of study entry, median (IQR)	1990 (1985-1997)	1990 (1984-1993)	1990 (1984-1992)	1990 (1986-1994)	1991 (1989-1997)
Year of birth, median (IQR)	1942 (1930-1952)	1944 (1932-1953)	1947 (1937-1958)	1949 (1940-1959)	1951 (1943-1959)
Birth country, No. (%)					
Born in SE, both parents born in SE	47 582 (92.0)	47 551 (92.0)	47 113 (91.1)	46 887 (90.7)	46 856 (90.6)
Born in SE, one/both parents born abroad	1659 (3.2)	1733 (3.4)	2409 (4.7)	2548 (4.9)	2721 (5.3)
Born abroad	2458 (4.8)	2415 (4.7)	2177 (4.2)	2264 (4.4)	2121 (4.1)
Population characteristics at last weight obs.					
Age (years), median (IQR)	52.8 (42.1-59.3)	50.2 (38.7-58.6)	45.9 (32.0-56.4)	44.7 (31.3-55.0)	45.6 (34.3-53.9)
Weight (kg), mean (SD)	72.4 (8.8)	76.6 (7.7)	79.5 (8.3)	83.4 (9.2)	93.2 (12.7)
Height (cm), mean (SD)	176.1 (6.7)	177.0 (6.3)	178.1 (6.2)	178.9 (6.2)	180.0 (6.4)
Body mass index (kg/m^2^), mean (SD)	23.3 (2.4)	24.5 (2.3)	25.1 (2.5)	26.1 (2.8)	28.8 (3.8)
Smoking status, No. (%)					
Never	21 400 (41.4)	21 636 (41.8)	22 032 (42.6)	22 192 (42.9)	20 804 (40.2)
Former	11 529 (22.3)	12 403 (24.0)	12 587 (24.3)	13 584 (26.3)	16 303 (31.5)
Current	17 715 (34.3)	16 421 (31.8)	15 494 (30.0)	14 591 (28.2)	13 870 (26.8)
No information	1055 (2.0)	1239 (2.4)	1586 (3.1)	1332 (2.6)	721 (1.4)
Marital status, No. (%)					
Unmarried	10 738 (20.8)	11 581 (22.4)	15 794 (30.5)	17 131 (33.1)	16 280 (31.5)
Married	35 054 (67.8)	34 965 (67.6)	31 285 (60.5)	30 027 (58.1)	30 218 (58.5)
Divorced or widow/-er	5907 (11.4)	5153 (10.0)	4620 (8.9)	4541 (8.8)	5200 (10.1)
Education level, No. (%)^b^					
Pre-upper secondary school <9 years	18 174 (35.2)	16 574 (32.1)	13 491 (26.1)	11 841 (22.9)	9998 (19.3)
Pre-upper secondary school 9 years	3922 (7.6)	3891 (7.5)	4474 (8.7)	4973 (9.6)	5640 (10.9)
Upper secondary school <3 years	15 953 (30.9)	17 339 (33.5)	19 482 (37.7)	20 713 (40.1)	21 993 (42.5)
Upper secondary school 3 years	6573 (12.7)	7025 (13.6)	7206 (13.9)	7144 (13.8)	6951 (13.4)
Post-upper secondary school ≥1 years	7077 (13.7)	6870 (13.3)	7046 (13.6)	7028 (13.6)	7116 (13.8)

Abbreviations: Obs. = observation/-s; IQR = interquartile range; SD = standard deviation; SE = Sweden.

a Predicted weight change values were derived from a linear mixed-effects model including random slopes for age. The mean and standard deviation (SD) reflect the average and variability of model-predicted weight change across individuals within each weight trajectory quintile.

b Highest educational attainment at the end of follow-up.

To examine PCa survival, we applied the 2-stage approach exclusively to PCa cases. Case-only Cox models were adjusted as in the full population, using age as the underlying timescale starting from PCa diagnosis. Additional adjustments included income (continuous), source of income (employed, unemployed, engaged in labor market policy activity, receiving economic aid, pensioner, early retiree, sick leave, no income), marital status, and Charlson comorbidity index (none, mild, moderate/severe),^35^ tumor aggressiveness at diagnosis for models of any PCa, as well as smoking status at the last weight observation. As the case-only analysis is less susceptible to detection bias after adjusting for tumor aggressiveness, we restricted the modelling of potential non-linear associations between weight trajectories and PCa death to the PCa case population. Natural cubic splines with 4 knots were employed to model non-linearity, which was assessed using a post-estimation Wald test to determine whether spline coefficients beyond the linear term jointly differed from zero. All analyses were conducted using Stata/MP version 18.0 (StataCorp LLC., College Station, Texas), with all tests being 2-sided and a significance level set at 0.05.

### Efforts to address potential sources of bias

To address information bias from subjectively reported weight, the mode of weight measurement was included as a predictor in the LME models. To explore detection bias, we conducted a period-stratified analysis of weight trajectories and incident PCa, comparing the pre-PSA era (before 1997) with the PSA era (1997 onwards). In the pre-PSA era, follow-up began at the last recorded weight and ended at PCa diagnosis, emigration, death, or December 31, 1996, whichever occurred first. In the PSA era, follow-up also began at the last weight observation, with delayed entry from January 1, 1997, and continued until diagnosis, emigration, death, or December 31, 2019. Within the PSA era, PCa cases were further stratified by detection mode: asymptomatic PSA testing versus symptom-driven detection (lower urinary tract symptoms [LUTS] or other symptoms). Although PSA testing was part of the diagnostic process in symptomatic cases, it was prompted by clinical symptoms rather than unrelated health-seeking behavior. For this analysis, follow-up commenced at the last weight observation, with delayed entry from January 1, 2000, when data on detection mode became available. As a sensitivity analysis, we included all men with at least 1 weight observation. Additionally, we applied a naïve survival model using weight change quintiles among men with weight observed before age 25 and after age 40. Annual weight change was calculated as the difference between the first and last weight observations divided by the number of years between them.

## Results

### Study population

**Figure 1.**
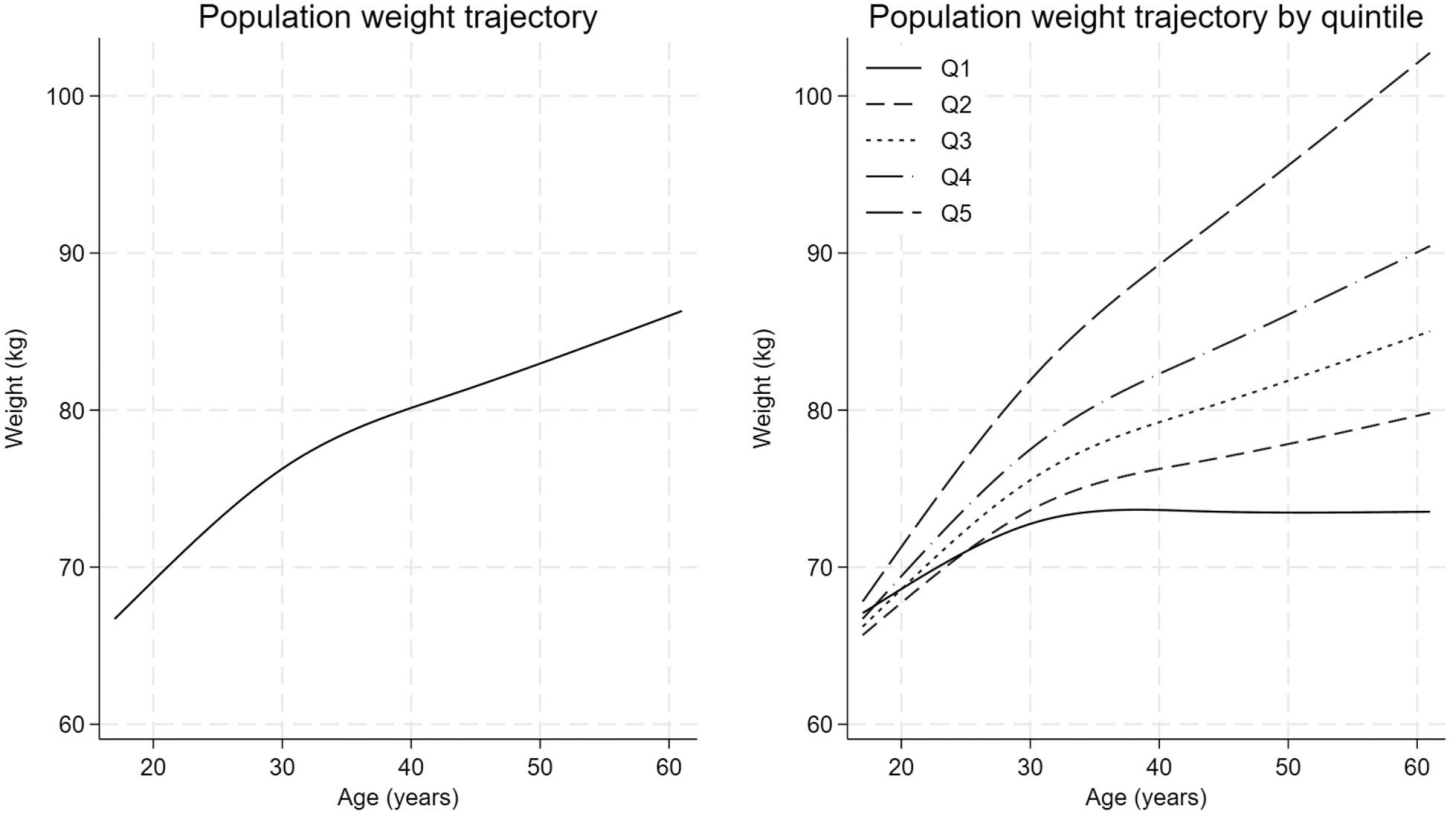
Adult weight trajectory by age for the population and by quintile. The population weight trajectory was estimated using a linear mixed-effects (LME) model with natural cubic splines for age and mode of weight measurement as predictors. The quintiles were categorized by the random weight slope. The weight trajectory by quintile shows the mean predicted weights by age in each quintile.

From ages 17 to 60, the mean weight gain was 19.6 kg. The average annual increase in weight was 0.73 kg/year (SD of random slope = 0.38) from 17 to <30 years, 0.34 kg/year (SD = 0.27) from 30 to <45 years, and 0.22 kg/year (SD = 0.21) from 45 to 60 years, indicating substantial between-individual variability in weight gain rates across age periods. Figure 1 illustrates weight trajectories across quintiles, with mean total weight gain from 6.5 kg in Q1 to 34.9 kg in Q5 (Table 1). Over a median follow-up of 25 years (IQR 14-30), 22 055 men (8.5%) were diagnosed with PCa at a mean age of 70 years (SD 8), and 4547 (1.8%) died from the disease. Clinical characteristics by PCa aggressiveness are detailed in Table S2 (see online supplementary material).

### Weight trajectories and prostate cancer outcomes

A steep weight gain trajectory was inversely associated with total PCa (HR for Q5 vs Q1 = 0.95, 95% CI = 0.91 to 0.99) and non-aggressive PCa (HR = 0.88, CI = 0.83 to 0.93), with no evidence of an association observed for aggressive PCa (HR = 1.03, CI = 0.95 to 1.13). In contrast, it was positively associated with PCa death (HR = 1.15, CI = 1.03 to 1.28) (Table 2). Weight gain from ages 17 to <30 years demonstrated a similar pattern, with inverse associations for total PCa (HR per 1 kg/year = 0.97, CI = 0.93 to 1.02) and non-aggressive PCa (HR = 0.90, CI = 0.85 to 0.94), and positive associations for aggressive PCa (HR = 1.11, CI = 1.01 to 1.23) and PCa death (HR = 1.20, CI = 1.04 to 1.39). Weight gain during later age periods did not follow the same clear pattern. Of note, a strong positive association with PCa death was observed for weight gain between ages 45 and 60 years (HR = 1.19, CI = 1.03 to 1.36).

**Table 2.** Hazard ratios (HRs) and 95% confidence intervals (CIs) of prostate cancer (PCa) incidence and death according to total and age-specific weight trajectories among 258 494 men in the Obesity and Disease Development Sweden study, 1963-2019.

	Any incident PCa (*n* = 258 494)		Incident non-aggressive PCa (*n* = 239 136)		Incident aggressive PCa (*n* = 239 136)		PCa death (*n* = 258 494)	
Weight trajectory quintile (ages 17 to 60)	No. events	HR (95% CI)^a^	No. events	HR (95% CI)^a^	No. events	HR (95% CI)^a^	No. events	HR (95% CI)^a^
Q1	5106	1.00	3243	1.00	1528	1.00	1218	1.00
Q2	5043	1.01 (0.97 to 1.05)	3309	1.02 (0.98 to 1.08)	1402	0.98 (0.91 to 1.05)	1138	1.02 (0.94 to 1.11)
Q3	4440	1.02 (0.98 to 1.06)	2933	1.01 (0.96 to 1.06)	1183	1.01 (0.93 to 1.09)	884	1.06 (0.97 to 1.15)
Q4	3959	0.98 (0.94 to 1.02)	2629	0.94 (0.89 to 0.99)	1052	1.00 (0.92 to 1.08)	752	1.13 (1.03 to 1.24)
Q5	3507	0.95 (0.91 to 0.99)	2331	0.88 (0.83 to 0.93)	931	1.03 (0.95 to 1.13)	555	1.15 (1.03 to 1.28)
Age period (per 1 kg/year)								
17 to <30 years	22 055	0.97 (0.93 to 1.02)	14 445	0.90 (0.85 to 0.94)	6096	1.11 (1.01 to 1.23)	4547	1.20 (1.04 to 1.39)
30 to <45 years		0.95 (0.90 to 1.00)		0.88 (0.83 to 0.94)		0.98 (0.88 to 1.09)		1.02 (0.89 to 1.17)
45 to 60 years		0.99 (0.93 to 1.06)		0.92 (0.85 to 1.00)		1.01 (0.90 to 1.15)		1.19 (1.03 to 1.36)

a The adult weight trajectories were estimated by linear mixed-effects (LME) models with natural cubic splines of age, and the age period weight trajectories by LME models with linear splines of age. Mode of measurement was included as predictor variable in the LME models. HRs were calculated from Cox regression models, stratified by birth cohort and adjusted for age, predicted weight at age 17, height, education, marital status, and birth country. The models for PCa death were additionally adjusted for smoking status, and the age period models for random weight slopes from the preceding age periods. For the models by aggressiveness, left truncation was applied from the start of the National Prostate Cancer Register, January 1, 1998. We defined aggressive PCa as T4 or N1 or M1 or Gleason score ≥8 or diagnostic PSA level of ≥50ng/mL, and other PCa cases as non-aggressive.

### PSA eras and mode of detection

The inverse association with PCa incidence was evident during the PSA era (HR Q5 vs. Q1 = 0.92, 95% CI = 0.87 to 0.97), particularly for asymptomatic PSA-detected cases (HR = 0.90, 95% CI = 0.84 to 0.96) (Table 3). No evidence of an association was observed for cases detected during the diagnostic work-up of LUTS or other symptoms. Similar trends were seen for early adulthood weight gain (ages 17 to <30).

**Table 3.** Hazard ratios (HRs) and 95% confidence intervals (CIs) of prostate cancer (PCa) incidence according to total and age-specific adult weight trajectories among men in the Obesity and Disease Development Sweden study (1963-2019), stratified by pre-PSA (1967-1996) and PSA (1997-2019) eras, and by detection mode within the PSA era

	Pre-PSA era 1967-1996 (*n* = 192 035)		PSA era 1997-2019 (*n* = 241 121)	
Weight trajectory quintile (ages 17 to 60)	No. cases	HR (95% CI)^a^	No. cases	HR (95% CI)^a^
Q1	555	1.00	4551	1.00
Q2	484	1.01 (0.89 to 1.14)	4559	1.01 (0.96 to 1.05)
Q3	354	1.06 (0.92 to 1.22)	4086	1.01 (0.96 to 1.05)
Q4	281	1.17 (1.01 to 1.37)	3678	0.95 (0.91 to 1.00)
Q5	149	1.04 (0.85 to 1.27)	3358	0.92 (0.87 to 0.97)
Age period (per 1 kg/year)				
17 to <30 years	1823	1.16 (0.95 to 1.41)	20 232	0.97 (0.92 to 1.01)
30 to <45 years		0.90 (0.69 to 1.19)		0.92 (0.87 to 0.98)
45 to 60 years		1.24 (0.97 to 1.60)		0.96 (0.90 to 1.03)

	PSA era 2000-2019 (*n* = 233 898)			
	Asymptomatic PSA-detected		Detection through LUTS or other symptoms	
Weight trajectory quintile (ages 17 to 60)	No. cases	HR (95% CI)^a^	No. cases	HR (95% CI)^a^
Q1	1752	1.00	2534	1.00
Q2	1818	1.01 (0.95 to 1.08)	2466	0.99 (0.94 to 1.05)
Q3	1754	1.01 (0.95 to 1.09)	2174	1.01 (0.95 to 1.07)
Q4	1700	0.98 (0.92 to 1.05)	1892	0.95 (0.89 to 1.01)
Q5	1559	0.90 (0.84 to 0.96)	1747	0.96 (0.90 to 1.02)
Age period (per 1 kg/year)				
17 to <30 years	8583	0.92 (0.86 to 0.98)	10 813	1.00 (0.93 to 1.07)
30 to <45 years		0.91 (0.84 to 0.99)		0.94 (0.87 to 1.02)
45 to 60 years		0.96 (0.86 to 1.08)		0.97 (0.89 to 1.07)

Abbreviations: PSA = prostate specific antigen; LUTS = lower urinary tract symptoms.

a The adult weight trajectories were estimated by linear mixed-effects (LME) models with natural cubic splines of age, and the age period weight trajectories by LME models with linear splines of age. Mode of measurement was included as predictor variable in the LME models. HRs were calculated from Cox regression models, stratified by birth cohort and adjusted for age, predicted weight at age 17, height, education, marital status, and country of birth. The age period models were additionally adjusted for random weight slopes from the preceding age periods. The pre-PSA era model was right-truncated on 31 December 1996, while the PSA era model was left-truncated on 1 January 1997. For the PSA era model by detection mode, left truncation was applied from 1 January 2000, when information on detection mode became available from the National Prostate Cancer Register.

### Weight trajectories and prostate cancer survival

**Figure 2.**
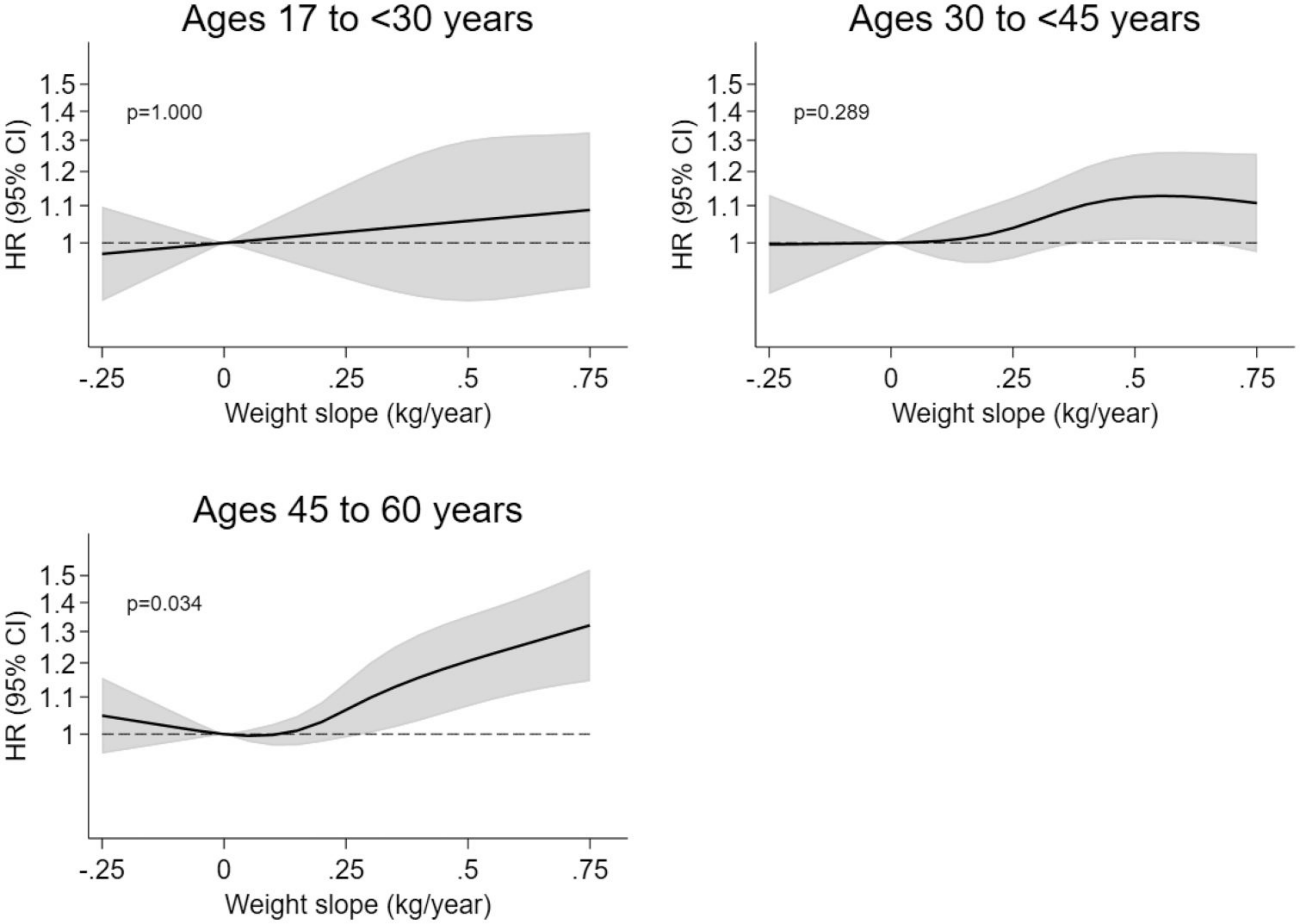
Hazard ratios (HRs) and 95% confidence intervals (CIs) of prostate cancer death among diagnosed cases, according to adult age-specific weight trajectories. The age period weight trajectories were estimated by linear mixed-effects (LME) models with linear splines of age and mode of measurement as predictors. Cox proportional hazards regression models with natural cubic splines were used to examine the non-linear association between weight slope and HR with 95% CI for PCa death. The *P*-value shows statistical evidence for non-linearity. All survival models were stratified by birth cohort and adjusted for age at diagnosis, predicted weight at age 17, random weight slopes from the preceding age periods, aggressiveness, height, smoking status, education, income, source of income, marital status, birth country, and Charlson comorbidity index.

Among 20 541 men with PCa, steep prediagnostic weight gain was positively associated with PCa death (HR Q5 vs. Q1 = 1.23, 95% CI = 1.08 to 1.40), particularly for weight gain occurring between ages 45 and 60 (HR per 1 kg/year = 1.31, 95% CI = 1.10 to 1.57) (Table 4). These associations were consistent irrespective of disease aggressiveness at diagnosis. In case-only analyses, weight gain during ages 45 to 60 demonstrated a non-linear association with PCa death, with a positive association emerging from approximately 0.15 kg/year (equivalent to a 2.25 kg gain over 15 years), progressing in a steeper pattern than observed in earlier age periods (Figure 2).

**Table 4.** Hazard ratios (HRs) and 95% confidence intervals (CIs) of prostate cancer (PCa) death according to total and age-specific adult weight trajectories among 20 541 PCa cases in the Obesity and Disease Development Sweden study, 1963-2019.

Weight trajectory quintile (ages 17 to 60)	PCa death in men with any PCa at diagnosis (*n* = 20 541)		PCa death in men with non-aggressive PCa at diagnosis (*n* = 14 445)		PCa death in men with aggressive PCa at diagnosis (*n* = 6 096)	
	No. deaths	HR (95% CI)^a^	No. deaths	HR (95% CI)	No. deaths	HR (95% CI)^a^
Q1	816	1.00	255	1.00	561	1.00
Q2	757	1.07 (0.97 to 1.18)	232	0.98 (0.82 to 1.17)	525	1.11 (0.98 to 1.25)
Q3	591	1.06 (0.95 to 1.18)	189	1.10 (0.90 to 1.33)	402	1.06 (0.93 to 1.21)
Q4	498	1.15 (1.02 to 1.29)	147	1.16 (0.94 to 1.43)	351	1.14 (0.99 to 1.31)
Q5	401	1.23 (1.08 to 1.40)	107	1.20 (0.94 to 1.52)	294	1.23 (1.06 to 1.44)
Age period (per 1 kg/year)						
17 to <30 years	3063	1.09 (0.94 to 1.26)	930	1.16 (0.88 to 1.51)	2133	1.04 (0.88 to 1.24)
30 to <45 years		1.13 (0.96 to 1.33)		1.14 (0.83 to 1.56)		1.14 (0.94 to 1.38)
45 to 60 years		1.31 (1.10 to 1.57)		1.36 (1.00 to 1.85)		1.25 (1.01 to 1.55)

a The adult weight trajectories were estimated by linear mixed-effects (LME) models with natural cubic splines of age, and the age period weight trajectories by LME models with linear splines of age. Mode of measurement was included as predictor variable in the LME models. HRs were calculated from Cox regression models, stratified by birth cohort and adjusted for age at diagnosis, predicted weight at age 17, height, smoking status, education, income, source of income, marital status, birth country, and Charlson comorbidity index. The models for PCa death among men with any PCa were additionally adjusted for disease aggressiveness, and the age period models for random weight slopes from the preceding age periods. The models exclude PCa cases before the start of the National Prostate Cancer Register, 1 January 1998. We defined aggressive PCa as T4 or N1 or M1 or Gleason score ≥8 or diagnostic PSA level of ≥50ng/mL, and other PCa cases as non-aggressive.

Sensitivity analysis using alternative definitions of weight trajectories and broader inclusion criteria yielded attenuated effect sizes but supported the same directional associations as the main analysis (Tables S3-S6—see online supplementary material).

## Discussion

In this large, nationwide pooled cohort of men in Sweden, weight gain across adulthood was inversely associated with non-aggressive PCa incidence and was not associated with aggressive PCa. In contrast, weight gain was positively associated with PCa death, particularly for weight accrued during early adulthood. Stratified analyses indicated that these associations with PCa incidence were confined to the PSA screening era and largely driven by asymptomatic PSA-detected cases. Case-only analyses further revealed that prediagnostic weight gain was linked to poorer survival from PCa, independent of tumor aggressiveness at diagnosis, with the strongest associations observed for weight gained during late middle adulthood.

Previous studies have reported similar inverse associations with non-aggressive PCa and positive associations with both aggressive PCa and PCa death.^7^^,^^17^^,^^19^^,^^36^ These opposing trends in the context of obesity and PCa have been widely debated and are likely to be relevant to our findings on weight trajectories.^9^^,^^37^^,^^38^ Detection bias is a plausible explanation for the inverse association with non-aggressive PCa observed in our data, as men of normal weight are more likely to undergo asymptomatic PSA testing, thereby increasing their likelihood of being diagnosed with non-aggressive PCa.^12^ Additionally, obesity is associated with lower PSA levels and prostate enlargement, which may delay diagnosis.^38^ To account for this, we stratified analyses by PSA era and detection mode, which showed that detection bias likely influenced PCa incidence estimates. As a result, opportunistic PSA testing limits the interpretability of associations between weight trajectories and PCa incidence, although the precise extent of its influence on our findings remains uncertain. A recent study based on a clinical cohort that accounted for screening practices suggested that additional mechanistic factors may be involved, as the inverse association with non-aggressive PCa was not accompanied by the expected positive association with aggressive PCa that would typically be anticipated in the presence of detection bias.^39^ In contrast, our case-only analysis, which is less susceptible to detection bias due to adjustment for disease aggressiveness at diagnosis, revealed a strong association between weight gain from ages 45 to 60 and PCa death. Collider stratification bias, a form of selection bias in case-only analyses, may arise when certain conditions are met, one being that the exposure is associated with inclusion in the analysis, ie, with PCa incidence in our case. The weak inverse association between weight gain and PCa incidence in our study suggests that any impact of collider bias on the observed association with PCa survival is likely to be small.^40^

To our knowledge, no prior studies have adjusted for weight trajectories across earlier life stages to isolate the associations of weight gain in late middle adulthood. Nonetheless, emerging evidence indicates that BMI at diagnosis is strongly associated with PCa survival.^41^ This suggests that biological mechanisms or differential treatment susceptibility may lie on the causal pathway linking obesity to PCa outcomes. Obesity influences several hormonal and metabolic pathways, characterized by elevated estradiol, insulin, free IGF-1, and leptin, and reduced free testosterone and adiponectin, all of which may contribute to PCa progression and are sensitive to weight change.^37^^,^^42^ However, the biological underpinnings of age-specific risk differentials in relation to weight trajectories remain poorly understood and warrant further investigation.^42^ We propose future research to examine the timing of obesity onset, particularly whether late-onset obesity poses a greater risk for PCa death than early-onset obesity. Such studies would help clarify whether obesity at diagnosis, rather than cumulative lifetime exposure, is a more critical determinant of PCa prognosis.

This study has several limitations. We were unable to account for unintentional weight loss and lacked data on PSA testing frequency and on potential confounding factors, such as physical activity and genetic profiling. The sample predominantly comprised ethnic Swedish men, which limits the generalizability to other populations. Moreover, joint modelling could have been a more parsimonious alternative to our 2-stage approach; however, it is currently too computationally intensive for the large number of observations in our study. This study also has notable strengths. It benefits from a large sample size, extended follow-up, and predominantly objectively measured weight data collected at multiple time points. Outcome ascertainment is based on high-quality registry linkages with detailed diagnostic and detection mode information. To our knowledge, this is the first study to examine individual weight trajectories across distinct adult age periods in relation to subsequent cancer outcomes. By adjusting for weight change in preceding intervals, our approach aligns with life course epidemiological recommendations for studying adiposity and cancer risk.^15^^,^^42^ Unlike group-based trajectory models, which overlook prior weight history, or single-point assessments that fail to capture longitudinal patterns, our method offers a comprehensive and temporally sensitive analytical framework. Furthermore, we introduce an interpretable metric: HRs per 1 kg/year weight gain over a 15-year interval, which may facilitate clearer interpretation in clinical and public health contexts.

### Conclusion

Our findings provide limited evidence for an association between adult weight gain and PCa incidence, highlighting the need for further research to identify risk factors beyond obesity. In contrast, case-only analyses demonstrated a strong association between weight gain in late middle adulthood and poorer PCa survival. These results suggest that maintaining weight stability during this life stage may contribute to improved PCa outcomes while also supporting broader health benefits.

## Supplementary Material

djag014_Supplementary_Data

## Data Availability

All data are hosted on Statistics Sweden’s Microdata Online Access (MONA) platform and may only be accessed from countries within the European Union or the European Economic Area. Access to data covered by the ethical approval will be considered in consultation with the principal investigator of the ODDS study, Tanja Stocks, and is subject to approval by the relevant register holders and the steering committees of the ODDS cohorts.
