# Supplementary material for: Weight trajectories throughout adulthood and prostate cancer incidence, aggressiveness, and death in 258 494 men
Source: J Natl Cancer Inst. 2026 Jan 23;118(6):1006–14. doi: 10.1093/jnci/djag014 (PMC13247337; doi:10.1093/jnci/djag014)
Supplement: djag014_Supplementary_Data [file djag014_supplementary_data.zip › 2025.10.30. Supplementary JNCI Revised.pdf]

Supplementary Material

Weight trajectories throughout adulthood and prostate cancer incidence, aggressiveness, and death in 258,494 men

Marisa da Silva, MSc, PhD[1,2]; Josef Fritz, MSc, PhD[1,3]; Ahmed Elhakeem, MPH, PhD[4,5]; Sylvia H. J. Jochems, MSc, PhD[6]; Ming Sun, MSc, PhD[1]; Innocent B. Mboya, MSc, PhD[1,7,8]; Christel Häggström, MSc, PhD[9]; Jens Wahlström, PhD[10]; Karl Michaëlsson, MD, PhD[11]; Patrik K. E. Magnusson, PhD[12]; Ylva T. Lagerros, MD, MPH, PhD[13]; Lena Lönnberg, PhD[14]; Abbas Chabok, MD, PhD[15]; Sölve Elmståhl, MD, PhD[16]; Bright I. Nwaru, MPhil, PhD[17,18]; Hannu Kankaanranta, MD, PhD[17,19,20]; Linnea Hedman, PhD[21]; Helena Backman, MSc, PhD[21]; Sara Hägg, MSc, PhD[12]; Pär Stattin, MD, PhD[22]; Kate Tilling, MSc, PhD[4,5]; Tanja Stocks, PhD[1]

[1]Department of Translational Medicine, Lund University, Malmö, Sweden; [2]School of Information Technology, Halmstad University, Halmstad, Sweden; [3]Institute of Clinical Epidemiology, Public Health, Health Economics, Medical Statistics and Informatics, Medical University of Innsbruck, Innsbruck, Austria; [4]Population Health Sciences, Bristol Medical School, University of Bristol, Bristol, UK; [5]MRC Integrative Epidemiology Unit, University of Bristol, Bristol, UK; [6]Institute for Risk Assessment Sciences, Utrecht University, Utrecht, The Netherlands; [7]Africa Academy for Public Health, Dar es Salaam, Tanzania; [8]Department of Epidemiology and Biostatistics, Institute of Public Health, Kilimanjaro Christian Medical University College, Moshi, Tanzania; [9]Northern Registry Centre, Department of Diagnostics and Intervention, Umeå University, Umeå, Sweden; [10]Department of Epidemiology and Global Health, Umeå University, Umeå, Sweden; University, Umeå, Sweden; [11]Medical Epidemiology, Department of Surgical Sciences, Uppsala University, Uppsala, Sweden; [12]Department of Medical Epidemiology and Biostatistics, Karolinska Institutet, Stockholm, Sweden; [13]Department of Medicine, Huddinge, Karolinska Institutet, Stockholm, Sweden; [14]Centre for Clinical Research, Västmanland, Uppsala university, Västerås, Sweden; [15]Department of Clinical Sciences, Division of Surgery, Danderyd Hospital, Karolinska Institutet, Stockholm, Sweden; [16]Department of Clinical Sciences Malmö, Lund University, Malmö, Sweden; [17]Krefting Research Centre, Department of Internal Medicine and Clinical Nutrition, Institute of Medicine, Sahlgrenska Academy, University of Gothenburg, Gothenburg, Sweden; [18]Wallenberg Centre for Molecular and Translational Medicine, University of Gothenburg, Gothenburg, Sweden; [19]Department of Respiratory Medicine, Seinäjoki Central Hospital, Seinäjoki, Finland; [20]Faculty of Medicine and Health Technology, Tampere University, Tampere, Finland; [21]Department of Public Health and Clinical Medicine, The OLIN and Sunderby Research Unit, Umeå University, Umeå, Sweden; [22]Department of Surgical Sciences, Uppsala University, Uppsala, Sweden

Corresponding Author: Marisa da Silva, MSc, PhD, Department of Translational Medicine, Lund University, Clinical Research Centre, Jan Waldenströms gata 35, 205 02, Malmö, Sweden (marisa.da_silva@med.lu.se).

Table of Contents

Table S1. Number and years of weight observations per cohort among 258,494 men in the Obesity and Disease Development Sweden study (1963–2019) 3

Table S2. Clinical characteristics of 20,541 men diagnosed with prostate cancer (PCa) in the Obesity and Disease Development Sweden study, 1963–2019 4

Table S3. Hazard ratios (HRs) and 95% confidence intervals (CIs) of prostate cancer (PCa) incidence and death according to total and age-specific adult weight trajectories among 2,089,144 men with one or more cancer-free weight observations in the Obesity and Disease Development Sweden study, 1963–2019 6

Table S4. Hazard ratios (HRs) and 95% confidence intervals (CIs) of prostate cancer (PCa) death according to total and age-specific adult weight trajectories among 54,237 PCa cases with one or more cancer-free weight observations in the Obesity and Disease Development Sweden study, 1963–2019 7

Table S5. Hazard ratios (HRs) and 95% confidence intervals (CIs) of prostate cancer (PCa) incidence and death according to total and age-specific adult weight trajectories among 106,859 men whose first weight observation was before age 25 and last after age 40 in the Obesity and Disease Development Sweden study, 1963–2019 8

Table S6. Hazard ratios (HRs) and 95% confidence intervals (CIs) of prostate cancer (PCa) death according to adult weight trajectories among 54,237 PCa cases whose first weight observation was before age 25 and last after age 40 in the Obesity and Disease Development Sweden study, 1963–2019 9

Figure S1. Flowchart of study participants 10

Table S1. Number and years of weight observations per cohort among 258,494 men in the Obesity and Disease Development Sweden study (1963–2019)

Cohort	Number of men[a]	Number of weight observations	Years of weight observations
**Construction Workers Cohort**	190,152	842,178	1971-1993
**Cohort of Swedish Men**	38,307	163,942	1938-2008[b]
**Swedish Military Conscription Register**	96,142	97,754	1969-2014
**Northern Sweden Health and Disease Study**	23,062	50,044	1985-2019
**Swedish Twin Registry**	9,268	19,415	1936-2013[b]
**Malmö cohorts[c]**	6,874	17,757	1944-2019[b]
**Swedish National March Cohort**	6,609	15,105	1938-1997[b]
**Westmannia Cardiovascular Risk Factors Study**	6,647	6,647	1989-2000
**EpiHealth**	3,574	6,353	1958-2018[b]
**West Sweden Asthma Study**	2,741	4,856	2008-2016
**Obstructive Lung Disease in Northern Sweden**	854	1,328	1986-2016
**LifeGene**	642	642	2009-2017
**Total**		1,226,021	1936-2019

[a]Includes overlaps between cohorts. That is, a man recorded in multiple cohorts is counted in all these cohorts, making the sum of the column (n=384,872) exceeding the total number of men in the study (n=258,494).
[b]Years before 1963 correspond to ages for which weight was recalled. The earliest year of weight data collection was 1963.
[c]Includes the Malmö Preventive Project, the Malmö Diet and Cancer Study, and the Malmö Offspring Study.

Table S2. Clinical characteristics of 20,541 men diagnosed with prostate cancer (PCa) in the Obesity and Disease Development Sweden study, 1963–2019

Characteristic	Any PCa (n=20,541)	Non-aggressive PCa[a] (n=14,445)	Aggressive PCa[a] (n=6,096)
**Age at diagnosis (years), mean (SD)**	69.5 (8.2)	68.1 (7.7)	72.9 (8.3)
**Age category, n (%)**			
**<65**	6,209 (30.2)	5,096 (35.3)	1,113 (18.3)
**65-70**	4,841 (23.6)	3,725 (25.8)	1,116 (18.3)
**70-75**	4,154 (20.2)	2,831 (19.6)	1,323 (21.7)
**≥75**	5,337 (26.0)	2,793 (19.3)	2,544 (41.7)
**Charlson comorbidity index, n (%)[b]**			
**0 (no comorbidity)**	14,172 (69.0)	10,446 (72.3)	3,726 (61.1)
**1 (mild comorbidity)**	3,236 (15.8)	2,193 (15.2)	1,043 (17.1)
**≥2 (severe comorbidity)**	3,133 (15.3)	1,806 (12.5)	1,327 (21.8)
**Mode for PCa detection, n (%)**			
**Asymptomatic health check-up**	8,579 (41.8)	6,796 (47.0)	1,783 (29.2)
**Lower urinary tract symptoms (LUTS)**	5,906 (28.8)	3,945 (27.3)	1,961 (32.2)
**Other symptoms**	4,791 (23.3)	2,853 (19.8)	1,938 (31.8)
**Missing**	1,265 (6.2)	851 (5.9)	414 (6.8)
**Local clinical tumour stage, n (%)**			
**T1a–b**	853 (4.2)	763 (5.3)	90 (1.5)
**T1c**	8,910 (43.4)	7,880 (54.6)	1,030 (16.9)
**T2**	6,282 (30.6)	4,505 (31.2)	1,777 (29.2)
**T3–4**	3,673 (17.9)	1,231 (8.5)	2,442 (40.1)
**Missing**	823 (4.0)	66 (0.5)	757 (12.4)
**Lymph node metastasis, n (%)**			
**N0**	4,552 (22.2)	3,054 (21.1)	1,498 (24.6)
**N1**	725 (3.5)	0 (0.0)	725 (11.9)
**Nx/missing**	15,264 (74.3)	11,391 (78.9)	3,873 (63.5)
**Bone metastasis, n (%)**			
**M0**	12,140 (59.1)	8,907 (61.7)	3,233 (53.0)
**M1**	1,710 (8.3)	0 (0.0)	1,710 (28.1)
**Mx/missing**	6,691 (32.6)	5,538 (38.3)	1,153 (18.9)
**Gleason score, n (%)**			
**≤6**	8,410 (40.9)	8,081 (55.9)	329 (5.4)
**7**	6,968 (33.9)	5,843 (40.4)	1,125 (18.5)
**8–10**	4,055 (19.7)	0 (0.0)	4,055 (66.5)
**Gx/missing**	1,108 (5.4)	521 (3.6)	587 (9.6)
**PSA at diagnosis, n (%)**			
**<4 ng/ml**	1,925 (9.4)	1,773 (12.3)	152 (2.5)
**4–9 ng/ml**	8,846 (43.1)	7,841 (54.3)	1,005 (16.5)
**10–49 ng/ml**	6,862 (33.4)	4,754 (32.9)	2,108 (34.6)
**≥50 ng/ml**	2,908 (14.2)	77 (0.5)	2,831 (46.4)
**Cancer risk category, n (%)[c]**			
**Localised low-risk**	5,883 (28.6)	5,883 (40.7)	0 (0.0)
**Localised intermediate-risk**	6,216 (30.3)	6,216 (43.0)	0 (0.0)
**Localised high-risk**	4,702 (22.9)	2,346 (16.2)	2,356 (38.6)

Characteristic	Any PCa (n=20,541)	Non-aggressive PCa[a] (n=14,445)	Aggressive PCa[a] (n=6,096)
**Regionally metastatic/locally advanced**	1,293 (6.3)	0 (0.0)	1,293 (21.2)
**Distant metastases**	2,447 (11.9)	0 (0.0)	2,447 (40.1)

SD: standard deviation.

[a]Aggressive PCa: T4 or N1 or M1 or Gleason score ≥8 or diagnostic PSA level of ≥50ng/mL; non-aggressive PCa: all other PCa cases.

[b]According to discharge diagnoses in the Swedish Patient Register.

[c] Localised low-risk: T1–2, Gleason score 2–6, and PSA <10 ng/ml; localised intermediate-risk: T1–2, Gleason score 7, and/or PSA 10 to <20 ng/ml; localised high-risk: T3 and/or Gleason score 8–10 and/or PSA 20 to <50 ng/ml; regionally metastatic/locally advanced: T4 and/or N1 and/or PSA 50 to <100 ng/ml in the absence of distant metastases; distant metastases: M1 and/or PSA ≥100 ng/ml.

Table S3. Hazard ratios (HRs) and 95% confidence intervals (CIs) of prostate cancer (PCa) incidence and death according to total and age-specific adult weight trajectories among 2,089,144 men with one or more cancer-free weight observations in the Obesity and Disease Development Sweden study, 1963–2019

	**Any incident PCa (n=2,089,144)**		**Incident non-aggressive PCa (n=1,987,056)**		**Incident aggressive PCa (n=1,987,056)**		**PCa death (n=2,089,144)**	
**Weight trajectory quintile (ages 17 to 60)**	No. events	HR (95% CI)[a]	No. events	HR (95% CI)[a]	No. events	HR (95% CI)[a]	No. events	HR (95% CI)[a]
**Q1**	18,219	1.00	11,586	1.00	5,464	1.00	4,626	1.00
**Q2**	9,242	1.06 (1.03-1.09)	5,940	1.03 (1.00-1.07)	2,027	1.09 (1.03-1.15)	1,376	1.07 (1.01-1.14)
**Q3**	8,571	1.07 (1.04-1.10)	6,043	1.06 (1.03-1.10)	1,609	1.04 (0.98-1.10)	865	1.05 (0.98-1.14)
**Q4**	8,432	1.02 (0.99-1.05)	6,069	1.00 (0.97-1.04)	1,686	1.02 (0.97-1.09)	804	1.04 (0.96-1.12)
**Q5**	14,932	0.98 (0.96-1.00)	10,330	0.94 (0.91-0.97)	3,493	1.00 (0.96-1.05)	2,154	1.11 (1.05-1.17)
**Age period (per 1 kg/year)**								
**17 to <30**		0.98 (0.95-1.01)		0.91 (0.88-0.95)		1.03 (0.96-1.10)		1.15 (1.03-1.28)
**30 to <45**	59,396	0.96 (0.92-1.01)	39,968	0.89 (0.84-0.94)	14,279	0.95 (0.87-1.05)	9,825	1.05 (0.93-1.19)
**45 to 60**		1.00 (0.94-1.06)		0.90 (0.84-0.98)		0.97 (0.87-1.09)		1.20 (1.06-1.37)

[a]The adult weight trajectories were estimated by linear mixed-effects (LME) models with natural cubic splines of age, and the age period weight trajectories by LME models with linear splines of age. Mode of measurement was included as predictor in the LME models. HRs were calculated from Cox regression models, stratified by birth cohort and adjusted for age, predicted weight at age 17, height, education, marital status, and birth country. The models for PCa death were additionally adjusted for smoking status, and the age period models for random weight slopes from the preceding age periods. For the models by aggressiveness, left truncation was applied from the start of the National Prostate Cancer Register, 1 January 1998. We defined aggressive PCa as T4 or N1 or M1 or Gleason score ≥8 or diagnostic PSA level of ≥50ng/mL, and other PCa cases as non-aggressive.

Table S4. Hazard ratios (HRs) and 95% confidence intervals (CIs) of prostate cancer (PCa) death according to total and age-specific adult weight trajectories among 54,237 PCa cases with one or more cancer-free weight observations in the Obesity and Disease Development Sweden study, 1963–2019

	**PCa death in men with any incident PCa (n=54,237)**		**PCa death in men with non-aggressive PCa at diagnosis (n=39,961)**		**PCa death in men with aggressive PCa at diagnosis (n=14,276)**	
**Weight trajectory quintile (ages 17 to 60)**	No. deaths	HR (95% CI)[a]	No. deaths	HR (95% CI)[a]	No. deaths	HR (95% CI)[a]
**Q1**	2,886	1.00	842	1.00	2,044	1.00
**Q2**	755	0.99 (0.92-1.07)	185	0.95 (0.82-1.11)	570	1.01 (0.92-1.10)
**Q3**	530	0.99 (0.90-1.09)	130	1.02 (0.85-1.23)	400	0.98 (0.88-1.09)
**Q4**	530	1.05 (0.95-1.15)	116	1.11 (0.92-1.35)	414	1.02 (0.91-1.14)
**Q5**	1,398	1.11 (1.03-1.18)	385	1.25 (1.10-1.43)	1,013	1.06 (0.98-1.15)
**Age period (per 1 kg/year)**						
**17 to <30**		1.04 (0.92-1.18)		1.15 (0.87-1.52)		1.00 (0.87-1.15)
**30 to <45**	6,099	1.17 (1.01-1.36)	1,658	1.29 (0.97-1.70)	4,441	1.13 (0.95-1.34)
**45 to 60**		1.31 (1.11-1.55)		1.49 (1.11-2.00)		1.20 (0.98-1.47)

[a]The adult weight trajectories were estimated by linear mixed-effects (LME) models with natural cubic splines of age, and the age period weight trajectory by LME models with linear splines of age. HRs were calculated from Cox regression models. stratified by birth cohort and adjusted for age at diagnosis, predicted weight at age 17, height, smoking status, education, income, source of income, marital status, birth country, and Charlson comorbidity index. The model for PCa death among men with any PCa was additionally adjusted for aggressiveness, and the age period models for random weight slopes from the preceding age periods. The models exclude PCa cases before the start of the National Prostate Cancer Register, 1 January 1998. We defined aggressive prostate cancer as T4 or N1 or M1 or Gleason score ≥8 or diagnostic PSA level of ≥50ng/mL, and other PCa cases as non-aggressive.

Table S5. Hazard ratios (HRs) and 95% confidence intervals (CIs) of prostate cancer (PCa) incidence and death according to total and age-specific adult weight trajectories among 106,859 men whose first weight observation was before age 25 and last after age 40 in the Obesity and Disease Development Sweden study, 1963–2019

	**Any incident PCa (n=106,859)**		**Incident non-aggressive PCa (n=103,911)**		**Incident aggressive PCa (n=103,911)**		**PCa death (n=106,859)**	
**Weight trajectory quintile (ages 17 to 60)**	No. events	HR (95% CI)[a]	No. events	HR (95% CI)[a]	No. events	HR (95% CI)[a]	No. events	HR (95% CI)[a]
**Q1**	2,312	1.00	1,505	1.00	709	1.00	590	1.00
**Q2**	1,943	1.02 (0.96-1.08)	1,391	0.98 (0.91-1.06)	584	1.07 (0.95-1.19)	305	0.90 (0.78-1.03)
**Q3**	1,575	1.01 (0.94-1.08)	1,163	0.97 (0.89-1.05)	423	1.01 (0.89-1.15)	242	1.10 (0.94-1.28)
**Q4**	1,318	1.02 (0.95-1.10)	972	0.97 (0.89-1.05)	353	1.10 (0.96-1.26)	176	1.19 (1.00-1.42)
**Q5**	935	0.98 (0.90-1.06)	670	0.95 (0.86-1.04)	232	1.05 (0.90-1.23)	120	1.12 (0.92-1.37)

[a]HRs were calculated from Cox regression models, stratified by birth cohort and adjusted for age, weight at first observation, height, education, marital status, and birth country. The model for PCa death were additionally adjusted for smoking status. For the models by aggressiveness, left truncation was applied from the start of the National Prostate Cancer Register, 1 January 1998. We defined aggressive PCa as T4 or N1 or M1 or Gleason score ≥8 or diagnostic PSA level of ≥50ng/mL, and other PCa cases as non-aggressive.

Table S6. Hazard ratios (HRs) and 95% confidence intervals (CIs) of prostate cancer (PCa) death according to adult weight trajectories among 54,237 PCa cases whose first weight observation was before age 25 and last after age 40 in the Obesity and Disease Development Sweden study, 1963–2019

	**PCa death in men with any incident PCa (n=1,171)**		**PCa death in men with non-aggressive PCa at diagnosis (n=382)**		**PCa death in men with aggressive PCa at diagnosis (n=789)**	
**Weight trajectory quintile (ages 17 to 60)**	No. deaths	HR (95% CI)[a]	No. deaths	HR (95% CI)[a]	No. deaths	HR (95% CI)[a]
**Q1**	358	1.00	129	1.00	229	1.00
**Q2**	254	1.00 (0.85-1.18)	77	0.99 (0.74-1.32)	177	1.01 (0.82-1.24)
**Q3**	192	0.90 (0.75-1.08)	53	0.95 (0.68-1.31)	139	0.88 (0.70-1.10)
**Q4**	194	1.16 (0.97-1.40)	69	1.42 (1.05-1.93)	125	1.07 (0.84-1.34)
**Q5**	173	1.14 (0.94-1.39)	54	1.34 (0.96-1.87)	119	1.04 (0.81-1.32)

[a]HRs were calculated from Cox regression models, stratified by birth cohort and adjusted for age at diagnosis, weight at first observation, height, smoking status, education, income, source of income, marital status, birth country, and Charlson comorbidity index. The model for PCa death among men with any PCa was additionally adjusted for disease aggressiveness. The models exclude PCa cases before the start of the National Prostate Cancer Register, 1 January 1998. We defined aggressive prostate cancer as T4 or N1 or M1 or Gleason score ≥8 or diagnostic PSA level of ≥50ng/mL, and other PCa cases as non-aggressive.

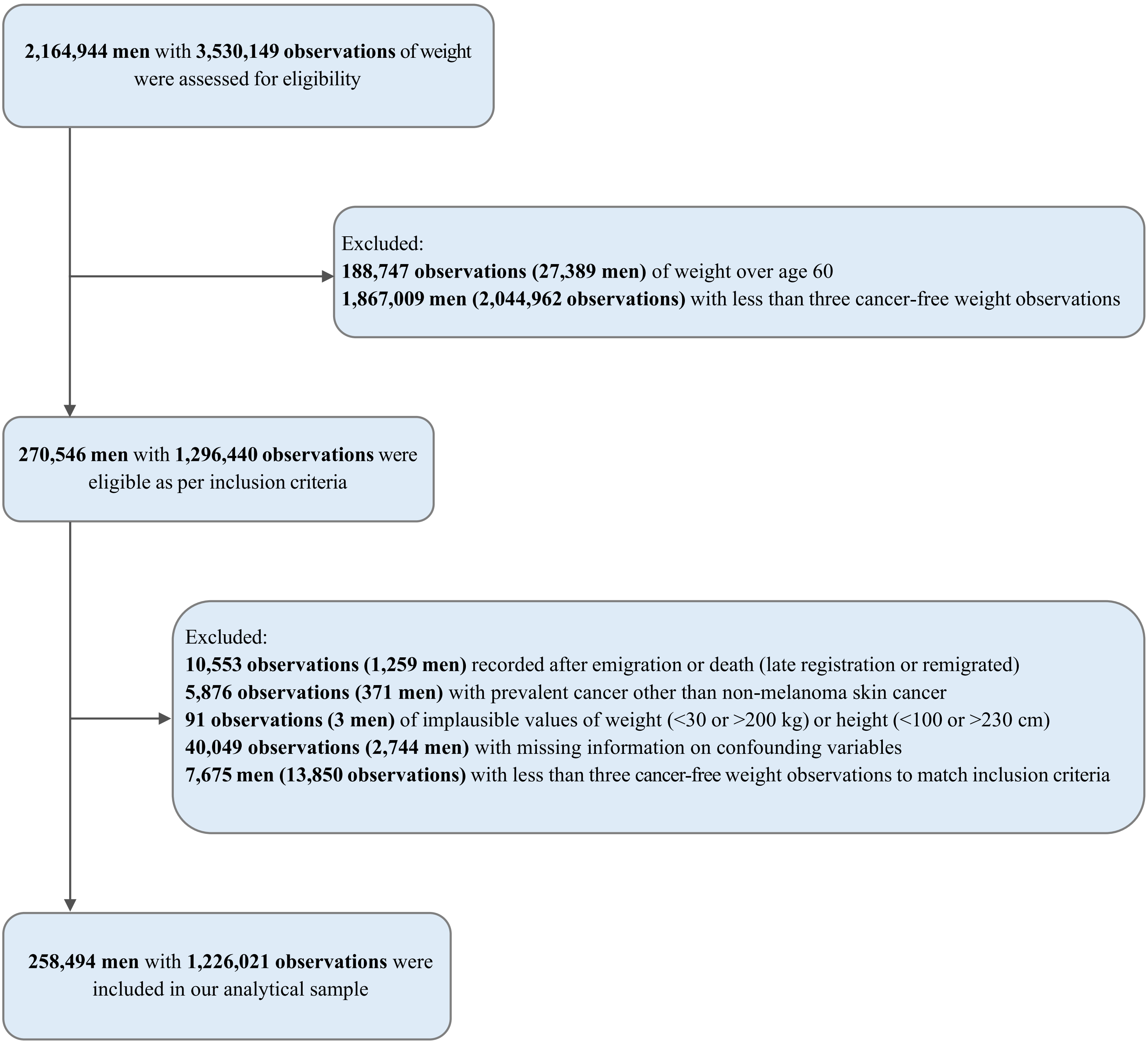

Figure S1. Flowchart of study participants
